# Production of aldosterone in cardiac tissues of healthy dogs and with dilated myocardiopathy

**DOI:** 10.14202/vetworld.2017.1329-1332

**Published:** 2017-11-12

**Authors:** Alejandro Reynoso-Palomar, Georgina Mena-Aguilar, Marisol Cruz-García, César Pastelín-Rojas, Abel Villa-Mancera

**Affiliations:** Faculty of Veterinary Medicine and Animal Husbandry, Meritorious Autonomous University of Puebla, Tecamachalco Puebla, México

**Keywords:** cardiac insufficiency, dog heart, enzyme-linked immunosorbent assay, mineralocorticoids

## Abstract

**Background and Aim:**

Aldosterone is a hormone, belonging to the group of mineralocorticoids, mainly synthesized in the adrenal cortex, basically its function is to regulate blood pressure and sodium-potassium levels in the body; high levels of this hormone have harmful effects in the organism and mainly in the heart in chronic form. Dilated cardiomyopathy is a progressive disease of heart muscle that is characterized by ventricular chamber enlargement and contractile dysfunction, is one of the most common cardiac conditions in dogs of medium and large breeds. The aim of the study was to determine and quantify if a dog’s cardiac cells possess the capacity to synthesize aldosterone, as well as, the differences that appear between a healthy heart and with dilated myocardiopathy (DMC).

**Materials and Methods:**

Cardiac tissues were used from six healthy dogs and six with DMC. Enzyme-linked immunosorbent assay was performed to determine if the dog’s heart cells synthesized this mineralocorticoid in a similar way to rat, rabbit, and human tissues, as well as quantitative differences between the healthy heart and DMC.

**Results:**

In healthy dog hearts, aldosterone values were 62.5 pG for both the atria and right ventricle and 125 pG for the left ventricle. As for dog hearts’ with DMC, results were 125 pG in all four cavities.

**Conclusion:**

Both the healthy and DMC dog hearts produce aldosterone in all four cavities, observing that production increases in the atria and right ventricle of those hearts with DMC, as an intrinsic mechanism of cardiac remodeling.

## Introduction

In dogs, 95% of cardiac diseases are acquired with a predominance of mitral valve degeneration in small breeds and cardiomyopathy in larger ones [1-3]. It has been documented that in canine, human, and rat species, cardiac fibrosis is produced only due to an excess of aldosterone and a greater salt consumption than considered normal. The histological features of aldosterone-induced cardiac fibrosis may include proliferation of cardiac myocytes, fibroblasts, and intense perivascular inflammation [4,5].

Most of aldosterone’s pathophysiological effects have a course of several weeks and therefore, are most likely dependent on mineralocorticoid receptors. However, some inflammatory markers (tumor necrosis factor and ED-1) and collagen III deposition increases have been observed in some models of fibrosis induced by mineralocorticoids [6-9].

In previous studies, we determined the presence of aldosterone (Al) receptors in both healthy dog cardiac tissues and with dilated myocardiopathy (DMC), their distribution, biochemical characteristics and the modifications present under both conditions [10,11]. In this research, we will determine if dog cardiac cells possess the capacity to synthesize this mineralocorticoid in a similar way as rat, rabbit [12-13] and human[14] tissues do, as well as, the differences that appear between the healthy heart and with DMC.

The aim of the study was to determine and quantify if a dog’s cardiac cells possess the capacity to synthesize aldosterone, as well as, the differences that appear between a healthy heart and with dilated myocardiopathy (DMC).

## Materials and Methods

### Ethical approval

To accomplish this research, tissues from the four cardiac chambers were subtracted from six healthy dogs and six dogs with DMC. These tissues had been used in a previously published study and kept in freezing [4], and approved by the Meritorious Autonomous University of Puebla Institutional Animal Care and use Committee (BCB-001/2012).

### Animals

Before euthanasia and their extraction from the rib cage, the heart samples were washed with Hartman’s solution with the intention of eliminating by drag, any blood trace including aldosterone of extracardiac origin. After thawing the cardiac tissues, homogenates, and enzyme-linked immunosorbent assay (ELISA) were performed to such, using a commercially specific kit (Cayman Chem, Aldosterone ELISA kit, Item #501090). Samples were read using an ELISA reader and a Bio Teck EL×800 plate washer and xChek Assay Management System software, version 3.20 Idexx Lab. Inc.

### Tissue homogenate procedure

About 100 mg of auricle and ventricle tissues were washed with 1×phosphate-buffered saline and homogenized with 1 mL of the same buffer using a homogenizer (Pro scientific Model Pro200) at a rate of 35,000 rpm and stored at − 20°C during 12 h. Two freeze-thaw cycles were performed to break the homogenate cell membranes; then they were converted into Eppendorf tubes and centrifuged for 5 min at 5000 g (6682 rpm) at − 4°C. The agglomerates were removed and the supernatants preserved at − 20°C in aliquots. Samples were centrifuged after being thawed and before being used (supernatants).

After completing the preparation of ELISA kit reagents, the aldosterone standard test was ran, with double dilutions in eight tubes as follows: In a standard tube, 100 μL of aldosterone was mixed with 900 μL of distilled water; from this tube, 100 μL of the contents were taken and added to 900 μL of distilled water, obtaining tube #1. From tube #1, 500 μL of its contents were taken and mixed to 500 μL of distilled water in another tube to obtain tube #2, and so on, the procedure is repeated until tube No 8. On completion of the aldosterone standard test, the samples were processed as follows:

The homogenates were thawed and centrifuged before their use; wells 1-A and B were left empty (Blk control), 150 μL of ELISA buffer was added to 50 μL of acetylcholinesterase (AchE) tracer (nonspecific binding [NSB] control) in wells 1-C and D.

In wells 1-E, F and G, a mixture of 100 μL ELISA buffer, 50 μL of AChE tracer and 50 μL of polyclonal aldosterone antiserum (control B0) were added. In the 1-H well, 5 μL of AchE tracer (TA control) were added. For wells 2-A to H, 50 μL of AChE tracer, 50 μL of antiserum and 100 μL of the aldosterone standard previously prepared (2-A well, tube #1, 2-B well, tube #2… 2-H well, tube #8) were added. In wells 3-6 A to H, 100 μL of the homogenates were added to 50 μL of AChE tracer and 50 μL of antiserum, incubated during 18 h at 4°C. The Ellman’s Reagent was reconstituted with distilled water, 5 wash cycles were performed to the plate with the buffer, and 200 μL of solution was added to the washed wells. In addition, another 5 μL of AChE tracings were mixed into the well with the TA control. Afterward, the plate was read at 405 nm using an ELISA reader (Biotek EL×800).

### ELISA

The assay was performed twice. Three plate readings were performed. The Blk control (1-A, B) values from both readings were averaged, this result was then subtracted from the value of each of the remaining wells of the plate, except the control values. The NSB and B0 control values were also averaged. The NSB average was subtracted from the B0 average to obtain the B0Max value. To graph the aldosterone curve values from the wells and samples, the following formula was used: Al value or sample - NSB/B0Max.

### Statistical analysis

Data were analyzed using the software IBM SPSS 20 for Windows (SPSS Inc., Chicago, USA). The Kolmogorov–Smirnov test was used to confirm normality. Results between groups were compared using the Student’s t-test when distributions were normal and the Mann–Whitney U-test when distributions were not. P ≤ 0.05 was considered significant.

## Results

**Figure-1:**
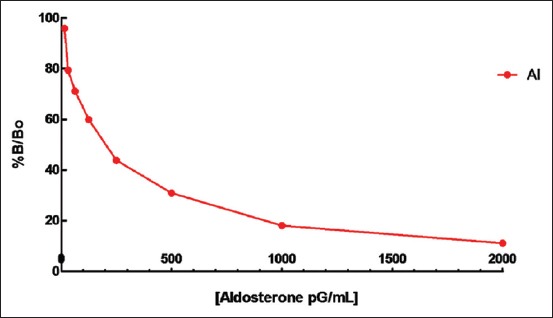
Aldosterone standard curve.

**Figure-2:**
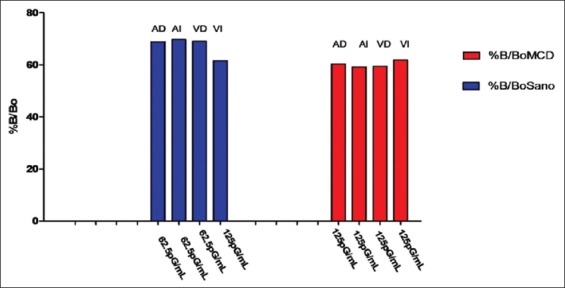
Average concentrations of intrinsic cardiac aldosterone.

Table-1 summarizes the aldosterone quantification per cardiac cavity in dogs. Both, the healthy dog heart as well as the heart with malonyl-CoA decarboxylase produces Al in all four cavities. It can also be observed that in the DMC dog heart, there is an aldosterone increase present in the atria and right ventricle, while in the left ventricle, no significant differences were detected in both hearts (healthy and with DMC). In the following Figures-1 and 2, Al concentrations between cavities and healthy hearts and DMC, can be analyzed and compared against the standard curve.

**Table-1:** Aldosterone quantification per cardiac cavity in dogs, according to the ELISA assay absorbance.

[pG]	Al	S	MCD	Cavity
2000	11.12			
1000	18.03			
500	30.92			
250	43.86			
125	59.89	68.89	60.3	AD
62.5	71.15	69.80	59.23	AI
31.3	79.38	69.04	59.45	VD
15.6	95.9	61.57	61.93	VI

[]=Concentration values in picograms (pG/mL), Al=Values from aldosterone curve, S=Values for healthy hearts per cavity, MCD=Values of sick hearts (AD=Right atrium, AI=Left atrium, VD=Right ventricle, VI=Left ventricle), MCD=MalonylCoA decarboxylase

## Discussion

Delcayre *et al*.[13] describe that the discovery of the production of Aldo in a rat’s heart demonstrates the existence of an Aldo tissular system that is the presence in a cell or a group of cells of all the biochemical elements necessary to hormone synthesis and of its specific receptors making a local autocrine or paracrine action possible.

Intervention of aldosterone from cardiac origin in remodeling of the left ventricle in post-infarction has been evidenced very recently in the rat, reinforcing the idea that mineralocorticoids have functions, in physiologic as well as in pathologic conditions, in cardiovascular tissue. In the literature review performed to over 60 articles, including those cited here, no reports were found in which the production of Al was not quantified within the heart of any species, neither by cardiac cavity nor in DMC, which is a first for the present study.

Our results demonstrate that a canine’s heart, regardless of being healthy or with DMC, is able to synthesize aldosterone, coinciding with reported data in those of rat, rabbit [12], and human [13,14] heart tissues.

Although there are too few animals to provide any meaningful conclusions, that because of the limitations of the protectionist groups that makes difficult work experimentally with dogs, and it demonstrates lower aldosterone values in the sick heart compared to the healthy hearts, those findings show that: The dog cardiac tissues have the property of aldosterone production, and a tendency to increase the myocardiopathy.

The non-variability in the production of aldosterone between the left ventricle of the healthy dog’s heart and those with DMC remains unclear; however, it is possible that as the present study is an acute type, the modifications could be presented at a longer time to produce or induced the disease.

Aldosterone receptor expression or recognition sites in cardiac tissues are increased in DMC [11]. Hence, according to the results reported in this paper, there is a correlation between these cell recognition sites, in response to AI production increases in the heart, which makes us assume, that these increases, promote the process of cardiac remodeling.

## Conclusion

The quantification of intrinsic cardiac aldosterone allows us to provide an unreported knowledge of variations in the healthy heart and with DMC. In addition to the involvement of the RAAS in the pathogenesis of the disease (endocrine aldosterone), the activation of the atrial myocardium and right ventricle of the heart is confirmed, perhaps as reinforcement to this system (autocrine/paracrine aldosterone) or compensatory mechanism of cardiac remodeling. In either case, as fully demonstrated, increases heart failure.

## Authors’ Contributions

AV and AR: Conceived and designed the experiments. AV, AR, GM, CP, and MC: Performed the experiments. AV and MC: Analyzed the data AV, AR, and MC: Contributed reagents/materials/analysis tools AV and AR: Wrote the paper. All authors read and approved the final manuscript.
